# An N-Type Pseudo-Static eDRAM Macro with Reduced Access Time for High-Speed Processing-in-Memory in Intelligent Sensor Hub Applications

**DOI:** 10.3390/s23239329

**Published:** 2023-11-22

**Authors:** Subin Kim, Ingu Jeong, Jun-Eun Park

**Affiliations:** 1 LX Semicon, Seoul 06763, Republic of Korea; sbkim@lxsemicon.com; 2 Department of Electrical and Computer Engineering, Sungkyunkwan University, Suwon 16419, Republic of Korea; ingu578@g.skku.edu; 3 Department of Electronics Engineering, Chungnam National University, Daejeon 34134, Republic of Korea

**Keywords:** artificial intelligence (AI), processing-in-memory (PIM), gain-cell embedded DRAM (eDRAM), pseudo-static leakage compensation (PSLC), retention time, sensor hub

## Abstract

This paper introduces an n-type pseudo-static gain cell (PS-nGC) embedded within dynamic random-access memory (eDRAM) for high-speed processing-in-memory (PIM) applications. The PS-nGC leverages a two-transistor (2T) gain cell and employs an n-type pseudo-static leakage compensation (n-type PSLC) circuit to significantly extend the eDRAM’s retention time. The implementation of a homogeneous NMOS-based 2T gain cell not only reduces write access times but also benefits from a boosted write wordline technique. In a comparison with the previous pseudo-static gain cell design, the proposed PS-nGC exhibits improvements in write and read access times, achieving 3.27 times and 1.81 times reductions in write access time and read access time, respectively. Furthermore, the PS-nGC demonstrates versatility by accommodating a wide supply voltage range, spanning from 0.7 to 1.2 V, while maintaining an operating frequency of 667 MHz. Fabricated using a 28 nm complementary metal oxide semiconductor (CMOS) process, the prototype features an efficient active area, occupying a mere 0.284 µm^2^ per bitcell for the 4 kb eDRAM macro. Under various operational conditions, including different processes, voltages, and temperatures, the proposed PS-nGC of eDRAM consistently provides speedy and reliable read and write operations.

## 1. Introduction

**Figure 1:**
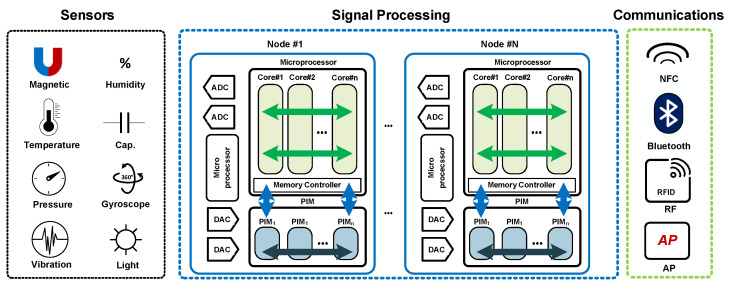
Conceptual structure of intelligent sensor hub system with sensors, communication interfaces, signal processing units, and processing-in-memory (PIM) units for accelerating neural network operations.

In recent years, extensive research has been conducted in the field of artificial intelligence by integrating semiconductors, big data, and machine learning algorithms. Notably, there is a growing focus on the advancement of efficient hardware solutions for Deep Neural Network (DNN) applications. Memory access constitutes a significant portion of the energy consumption in modern microprocessors, and the von Neumann architecture imposes limitations on throughput and latency in processing DNNs. To address this memory bottleneck, one prominent solution is the adoption of a processing-in-memory (PIM) or computing-in-memory (CIM) architecture, aimed at achieving optimized data processing. Figure 1 illustrates the structure of PIM in an intelligent sensor hub.

Recently, there has been a surge in the introduction of SRAM-based PIM solutions for energy-efficient DNN processing. The proposed PIM architecture, leveraging the SRAM bitcell, offers not only commendable processing speed but also logic compatibility [1,2,3,4]. Nonetheless, the SRAM bitcell encounters limitations due to its reduced integration potential arising from the bitcell size. Additionally, for ensuring stable multiply–accumulate (MAC) operations, supplementary transistors and bitlines become prerequisites [5,6]. 

Alternatively, ongoing research is exploring PIM architectures based on emerging non-volatile memories such as RRAM [7,8,9,10,11] and PCRAM [12], which offer features of compact form factors and high densities. Additionally, MRAM [13,14,15], which operates based on the magnetoresistance effect, also provides high density and non-volatility, along with fast read and write speeds, making it suitable for adoption in PIM structures with the potential to reduce power consumption. However, their limited applicability within the general CMOS process leads to increased manufacturing costs, usage of unstable resistors, and diminished energy efficiency.

**Figure 2:**
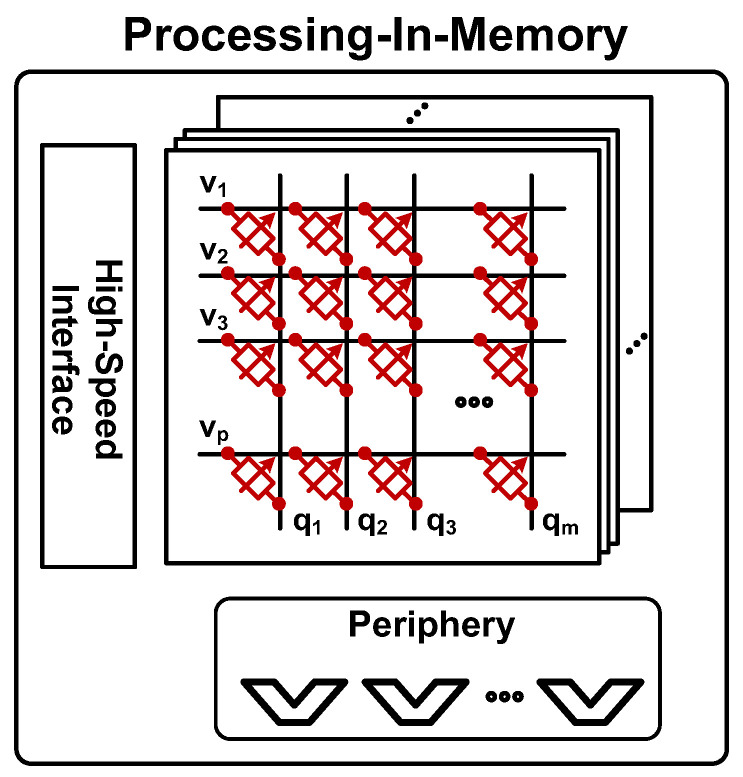
Conceptual block diagram of interface and periphery structures designed to handle MAC operation value processing in high-speed PIM architecture.

As an alternative, several studies have suggested PIM approaches based on embedded dynamic RAM (eDRAM) [16,17,18,19]. Compatible with general logic processes, eDRAM provides higher integration and smaller area compared to those of SRAM [20]. Therefore, eDRAM-based PIMs can realize more area-efficient implementation than SRAM-based PIMs. Figure 2 presents an illustrative depiction of the conceptual block diagram of a high-speed PIM configuration based on eDRAM.

Gain cell of eDRAM represents a structure that stores data in the storage node (SN). Consequently, it possesses a finite data retention time, attributed to leakage current, which necessitates periodic refresh to uphold stored data’s integrity [21,22,23]. The data retention time can be extended by applying an additional capacitor inside the gain cell [24,25]. However, large capacitors suffer from increased area and integration such as analog PIMs that require metal–oxide–metal (MOM) for MAC operation [18,19]. Additionally, when the structure of the same gain cell is migrated to processes below 28 nm, data retention time will be significantly reduced due to increased leakage current. The retention time of eDRAM can be extended using a specialized process rather than the conventional CMOS process. There are studies that proposed high integration using high-K/metal-gate technology [26] and extending retention time through TFT technology [27]. However, these approaches were constrained by the fact that they were not compatible with CMOS processes and required additional expensive processes.

This paper presents an eDRAM macro based on a pseudo-static n-type gain cell (PS-nGC), designed to ensure data retention even within miniaturized processes. The PS-nGC consists of a conventional 2T1C gain cell and a 3T n-type PSLC with pull-up logic, enabling active compensation for leakage current. As a result, the need for the capacitor, which is necessary in the conventional 2T1C configuration for data retention, can be eliminated, leading to enhanced efficiency in eDRAM area and density. Additionally, a summary and comparison between the PS-nGC and PS-pGC [28] are provided.

This paper is organized as follows. Section 2 describes the overview of eDRAM gain cell topologies and limitations of a prior work [28]. Section 3 explains the operating principle and circuit implementation of the proposed PS-nGC and eDRAM macro. Section 4 presents the simulation and experimental results. Section 5 presents a comparison between the proposed PS-nGC and PS-pGC in [28]. Finally, Section 6 presents the conclusions of this study.

## 2. Overview of eDRAM Gain Cell Topologies and Limitations of Previous Work

**Figure 3:**
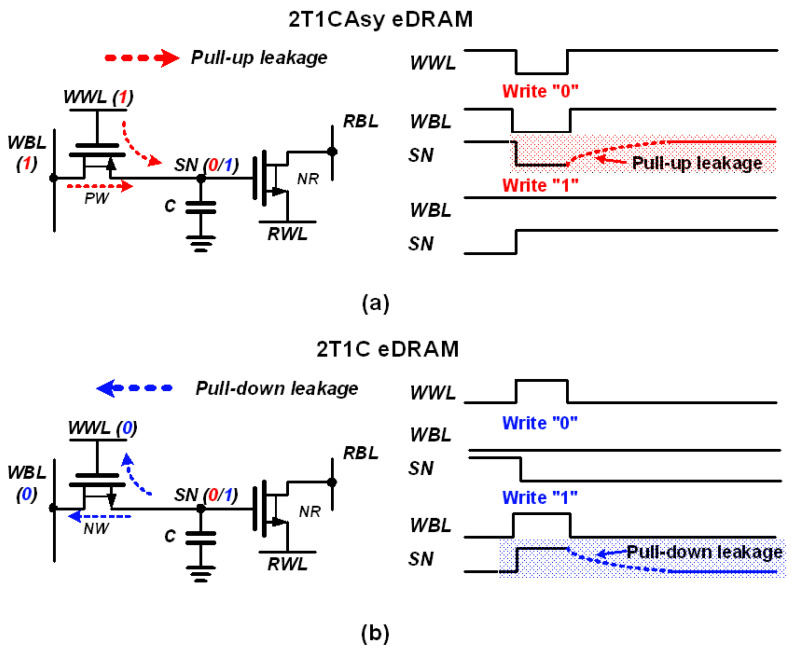
Leakage current paths in schematics of conventional (**a**) 2T1CAsy gain cell and (**b**) 2T1C gain cell and the timing diagrams of data flipping due to leakage current after write operation.

**Figure 4:**
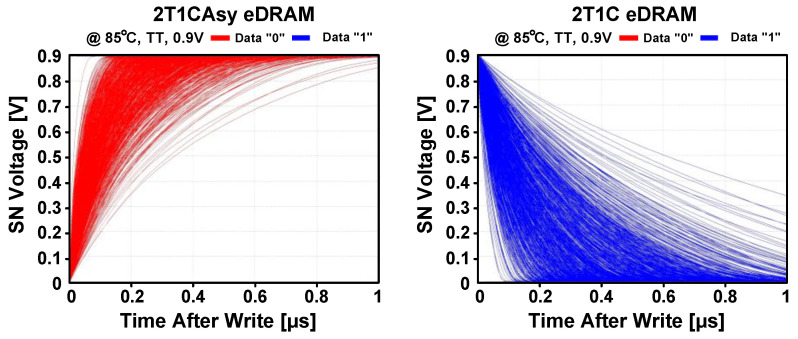
Monte Carlo simulations during data hold mode of 2T1CAsy and 2T1C gain cells for data “0” and “1” with 1000 trials.

Conventional 2T1C asymmetric (2T1CAsy) gain cell [29] and 2T1C gain cell [30] consists of two transistors and one capacitor as depicted in Figure 3. Data write operation is conducted by activating either the PW or NW transistor, transferring voltage from write bitline (WBL) to the internal SN. For data read operation, the NR transistor turns on to sense the stored data at the SN: either “0” or “1”. The retention time of the eDRAM gain cells primarily depends on two factors: total capacitance at the SN and leakage current by the PW or NW. For example, leakage current from the PW or NW can induce voltage fluctuation at the SN, leading to unintended data flipping. In the case of the 2T1CAsy gain cell [29], leakage current through the PW transistor increases the SN voltage, resulting in the data flipping from “0” to “1”. Conversely, in the 2T1C gain cell [30], leakage current through the NW transistor discharges the voltage at the SN, leading to data flipping from “1” to “0”. Figure 4 shows the data flipping issues in the conventional gain cell structures using Monte Carlo simulation. The data flipping arises from the leakage between the SN and write wordline (WWL) or WBL because WWL and WBL maintain the opposite voltages to the stored voltage at the SN, thereby affecting the retention time.

Various methods have been proposed to prevent the phenomenon where the voltage in the SN flips due to leakage current. For instance, under cryogenic conditions, the retention time can be extended due to its low leakage [31]. Additional bitlines or wordlines were employed to reduce the leakage current during data hold mode [24,25]. Another approach involves detecting retention failure and lowering the refresh cycle. However, even with these methods, the retention time remains finite, and refresh operations are still necessary.

**Figure 5:**
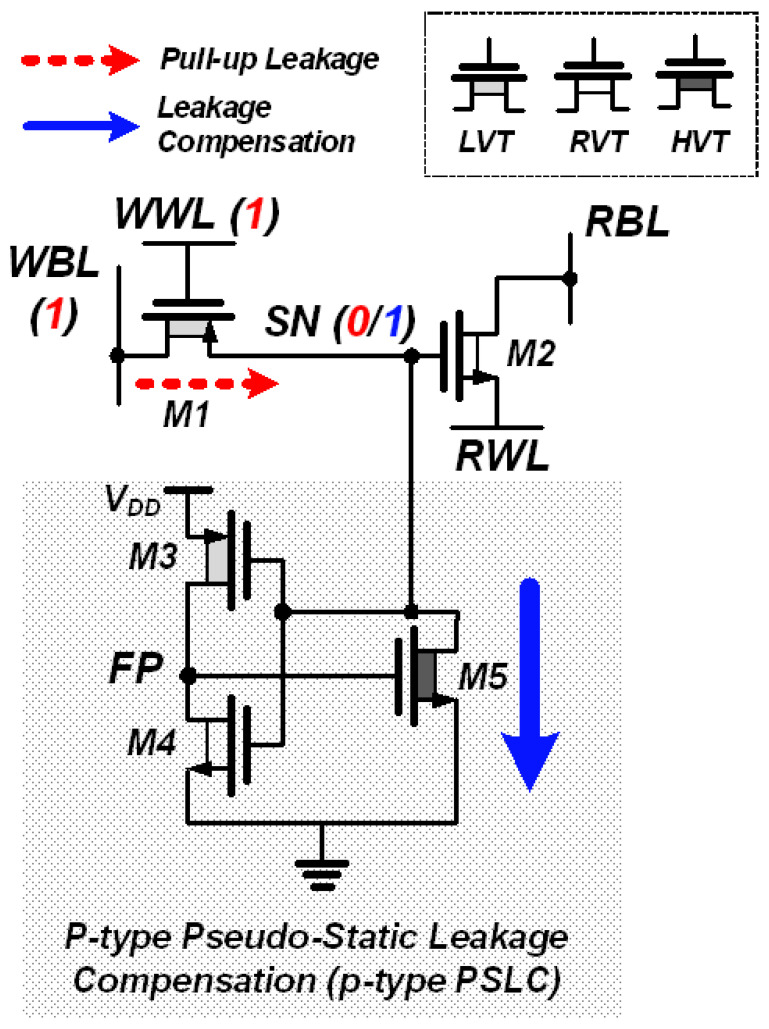
Schematic of PS-pGC with 2TAsy gain cell and p-type PSLC. Leakage current increases when data are “0” in the SN and p-type PSLC compensates the leakage current through M5.

**Figure 6:**
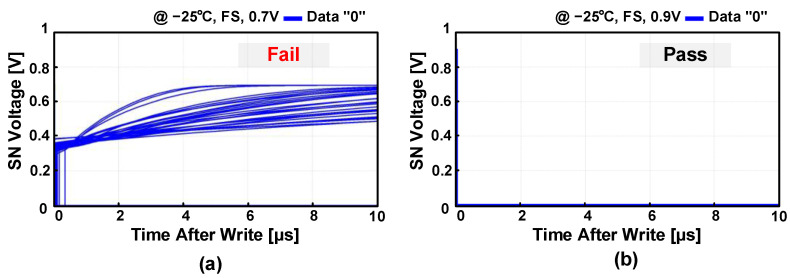
Monte Carlo simulations of SN voltage after data write operations of PS-pGC at supply voltage of (**a**) 0.7 V and (**b**) 0.9 V.

The previous work [28] presented the PS-pGC, which addressed the leakage current issue in the 2T1CAsy gain cell by actively compensating the leakage current. The PS-pGC consists of a 2TAsy gain cell and a p-type PSLC as shown in Figure 5. When storing data “0”, the p-type PSLC is activated, pulling down the leakage current and allowing SN to hold data “0” without voltage increase. The leakage compensation by the PS-pGC was able to extend retention time without use of an additional capacitor or power-hungry refresh operation in DRAM. While the PS-pGC offers a number of advantages as mentioned above, slow write access time due to PMOS transistor PW should be addressed in order to be adopted in the high-speed PIM applications. Figure 6 presents the Monte Carlo simulation results of the PS-pGC [28] after the write operation of data “0”. At the supply voltage of 0.7 V, which is lower than operating supply range, the PS-pGC fails to achieve successful write operation of data “0” due to too-low supply voltage. On the other hand, the PS-pGC successfully completed the write operation of data “0” at supply voltage of 0.9 V. When the PS-pGC writes data “0” to the SN, the worst-case scenario can result from the low supply voltage and the limitation imposed by the *V_TH_* of the PMOS M1, leading to slower activation. The improvement of write access time can be achieved in the 2TAsy structure by increasing the size of M1, but this approach comes with a trade-off where the increased cell area may lead to reduced access time. For example, increasing the width of M1 or M2 enhances transconductance, which can lead to faster write/read times. However, as the size of the transistor increases, parasitic capacitance also increases, ultimately leading to a potential decrease in bandwidth. Additionally, with an increasing number of bit cells integrated into the memory, the parasitic capacitance between the bit cell and the write/read bitline increases rapidly. Consequently, the speed of bit cell activation and the bandwidth of the sense amplifier for reading the bitline decrease, making it impractical to achieve fast read and write operations. Although employing a voltage-boosted WWL technique [32,33] can mitigate the issues, the active low operation of the PS-pGC makes it difficult to adopt the boosted WWL structure.

## 3. Operating Principle and Circuit Implementation of Proposed PS-nGC

**Figure 7:**
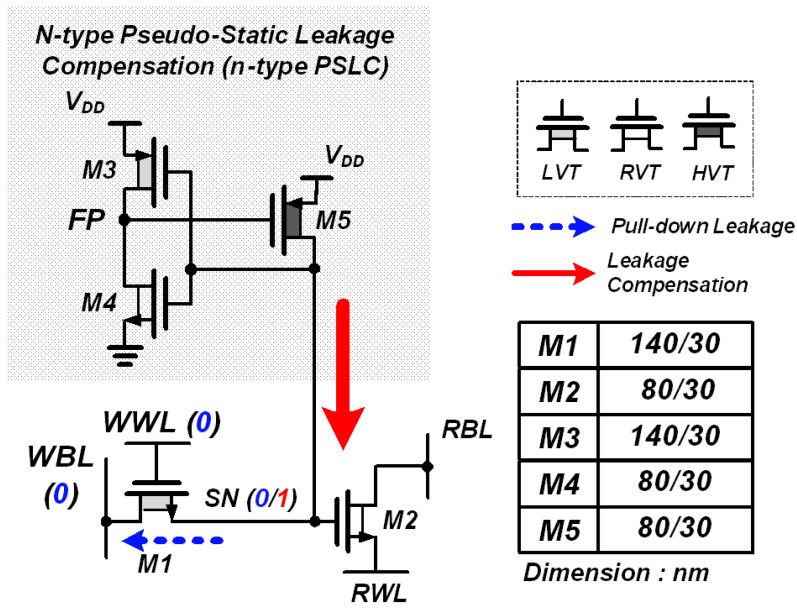
Schematic of proposed PS-nGC with 2T gain cell and n-type PSLC. Transistor dimensions of PS-nGC. Leakage current increases when data are “1” in the SN and n-type PSLC compensates the leakage current through M5.

The proposed PS-nGC is an eDRAM gain cell aimed at achieving fast access times and extended data retention without data loss issue. The PS-nGC comprises a 2T gain cell and an n-type PSLC with pull-up logic, as depicted in Figure 7. To improve the write access time, the PS-nGC employs an NMOS write access transistor M1 with higher charge mobility instead of the previously used PMOS write transistor in the PS-pGC [28]. Additionally, the application of a boosted voltage to WWL enables rapid activation of M1.

Because M1 was implemented with low *V_TH_* (LVT) transistor to reduce the write access time, the charge loss at the SN is dominated by M1 when storing data “1”. To address this issue, the n-type PSLC structure was designed to actively compensate for the charge loss during data “1” storage. The n-type PSLC structure includes an auxiliary inverter composed of M3 and M4 and compensation transistor M5. During the data hold operation, while storing data “1”, the auxiliary inverter activates M5 to supplement current for compensating the leakage current through M1. To minimize the subthreshold leakage current through M5 during the storage of data “0”, M5 was implemented with a high *V_TH_* (HVT) transistor, which helps to prevent unintentional data loss and to ensure efficient data storage for data “0”.

**Figure 8:**
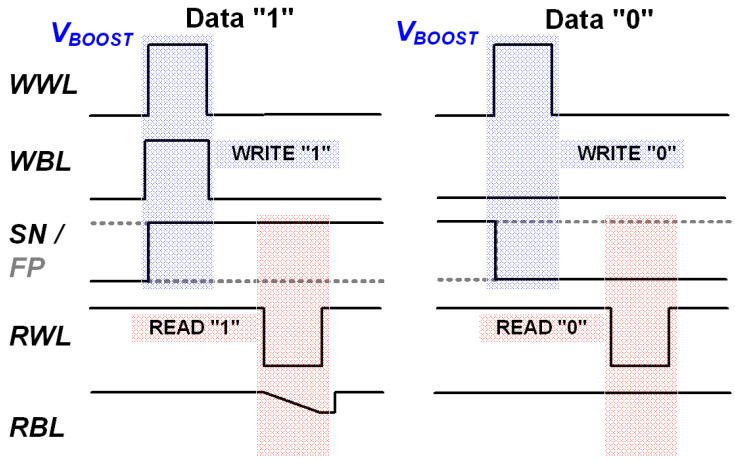
Conceptual timing diagram of PS-nGC write and read operations with boosted WWL when data are “0” and “1”, respectively.

Figure 8 illustrates the timing diagram for the read and write operations of data “0” and “1” in the PS-nGC. During the write operation, WWL is charged with *V_BOOST_* to reduce charge loss when transferring data “1” from WBL to the SN through M1. The voltage of WBL is charged to *V_DD_* only at writing data “1”. Otherwise, WBL remains at GND during hold or read operations. When data “1” is stored in the SN, the transistor M4 pulls down the FP node to GND, and the M5 turns on to compensate for the leakage current by the write/read access transistors M1 and M2. On the other hand, when “0” is stored in the SN, the M3 turns on, and the FP node is driven to *V_DD_*, maintaining the SN voltage to “0”. The read operation starts with discharging RWL to GND. Then, the voltage of RBL is determined by the data stored in the SN.

**Figure 9:**
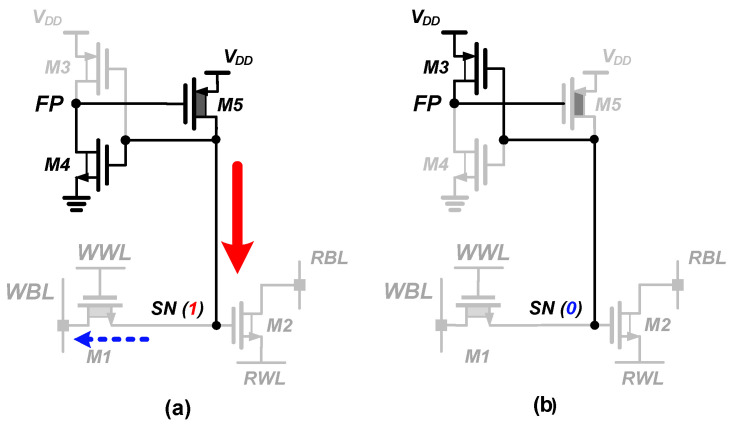
Leakage compensation principles of PS-nGC. (**a**) When storing data are “1”, compensation is activated. (**b**) When storing data are “0”, compensation circuit is deactivated.

Figure 9 shows the detailed operation of the n-type PSLC in the PS-nGC. When the SN stores data “1”, M4 is activated, forcing M5 to compensate for the leakage current. through M1 and M2. This ensures an extended retention time for storing data “1” and enables the pseudo-static operation of the 2T gain cell. On the other hand, when the SN stores data “0”, only M3 is turned on, and the FP node is driven to *V_DD_*. Consequently, M5 is turned off, maintaining the SN to retain its state. Because M5 is implemented with an HVT transistor, the leakage current during the deactivation is negligible compared to the charge injection through M1 or M2. As a result, the PS-nGC with the n-type PSLC can maintain its data without employing additional capacitors, which is similar to SRAM [34,35].

**Figure 10:**
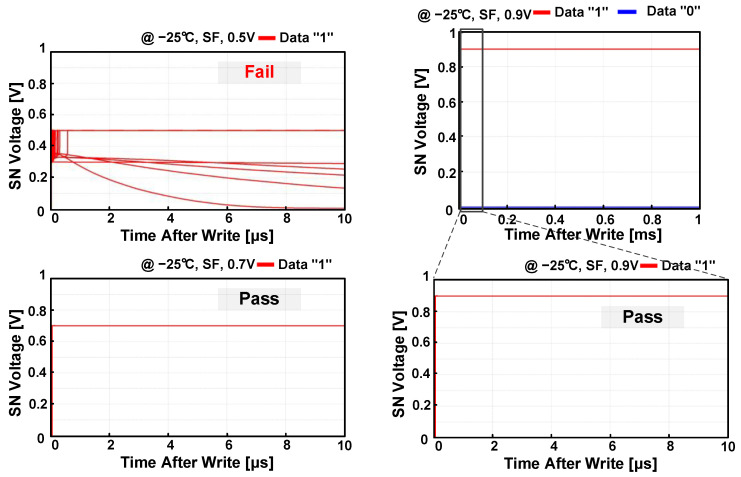
Monte Carlo mismatch simulations of data retention after writing data “1” with 1000 trials. At the worst operating condition, write operations fail at a supply of 0.5 V and success at supplies of 0.7 V and 0.9 V, respectively.

**Figure 11:**
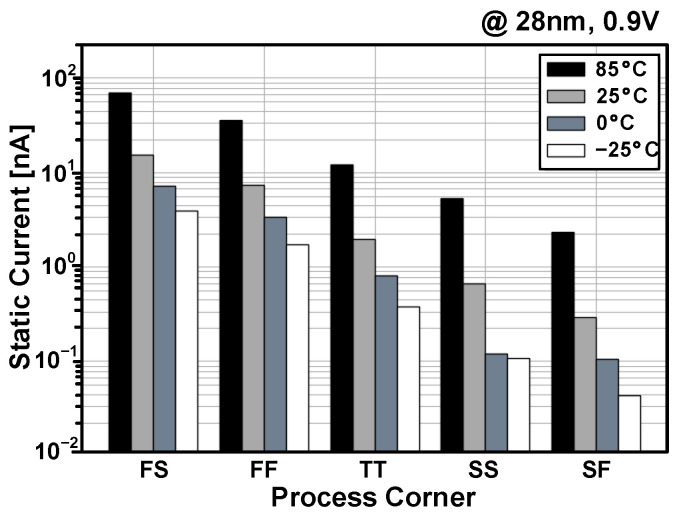
Post-layout simulated static currents of PS-nGC across five process corners and four temperature cases at a supply of 0.9 V.

Figure 10 shows the simulated SN voltage after writing data “1” using the Monte Carlo mismatch simulation. Compared with the fail operation at the supply voltage of 0.5 V, the PS-nGC successfully completed the writing operation at the supply voltage of 0.7 V, which is lower than the minimum supply voltage of the PS-pGC in Figure 6. After the writing of data “1”, the n-type PSLC was successfully activated, enabling the PS-nGC to preserve the stored voltage. Therefore, there is no change in the SN voltage observed until 1 ms after the write operation. Figure 11 illustrates the post-layout simulated static current of the PS-nGC during the hold operation with data “1”. The feedback configuration of the n-type PSLC allows for leakage compensation, ensuring a stable SN voltage regardless of process and temperature variations. The NMOS transistor M1 is the main source of leakage, resulting in the largest static current consumption at 85 °C in the FS and FF process corners.

**Figure 12:**
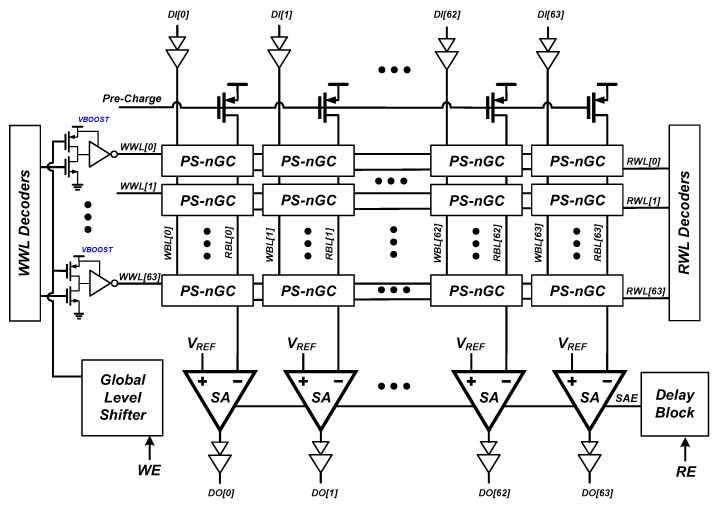
Overall architecture of 4 kb eDRAM macro consisting of 4 kb PS-nGC, WWL/RWL decoders, global level shifters for boosting WWL, and differential sense amplifiers.

**Figure 13:**
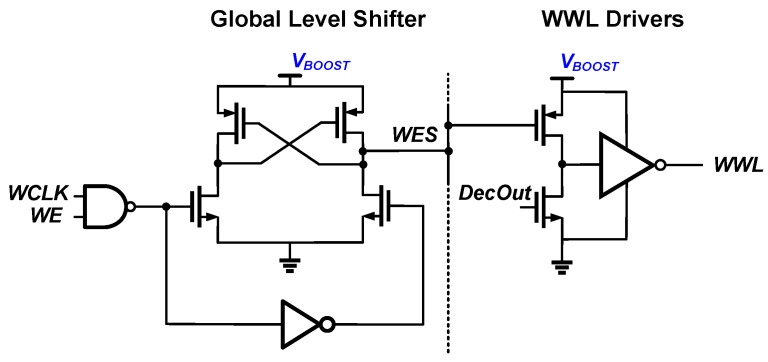
Global level shifter and WWL decoder to boost WWL driver output voltages to *V_BOOST_*.

Figure 12 shows the overall architecture of 4 kb eDRAM macro based on the proposed PS-nGC. The macro consists of a 4 kb (64 × 64) n-type PS-GC array, 64 differential sense amplifiers, and peripheral circuitry. The peripheral circuits consist of WWL driver decoder, RWL driver decoder, WBL driver, precharge driver, global level shifter, and delay block. The eDRAM cells in each row share WWL and RWL. Similarly, the eDRAM cells in each column share WBL and RBL. For the read operations, each RBL is precharged. After the precharging, the RWL decoder activates the read operation by driving an inverted pulse to each row. Then, the differential sense amplifiers of each column compare the voltages of each RBL with a reference voltage *V_REF_*. For the writing operation, the boosted control voltage *V_BOOST_* is applied to WWL [32,33]. Figure 13 shows a global level shifter and WWL driver circuit used for boosting the WWL signal. The WWL decoder signal is boosted to *V_BOOST_* using the global level shifter.

**Figure 14:**
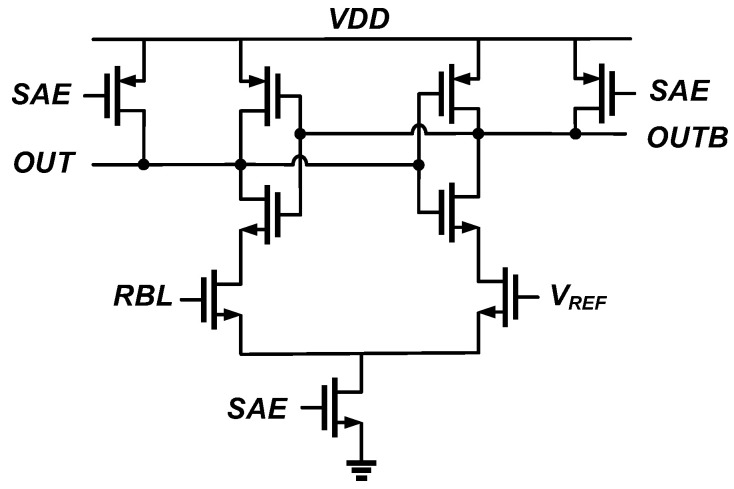
Schematic of differential sense amplifier for sensing voltage difference between RBL and *V_REF_* during read operation.

Prior eDRAMs [36,37,38,39] had popularly employed an inverter-based sense amplifier to detect the voltage of RBL during the read operation because of its compact implementation. However, the inverter-based sense amplifiers were prone to parasitic capacitance, resistance of the RBL, and leakage current by inactivated gain cells. To address these issues, this work employs a differential sense amplifier as shown in Figure 14. Before the read operation, the RBL is precharged to *V_DD_*. During the read operation, each RWL is activated, and the selected cells discharge the RBL depending on its stored data. At this time, a sense-amplifier enable (SAE) signal is activated, and the differential sense amplifier compares voltage of the discharged RBL with a reference voltage *V_REF_.*

## 4. Simulation and Experimental Results

**Figure 15:**
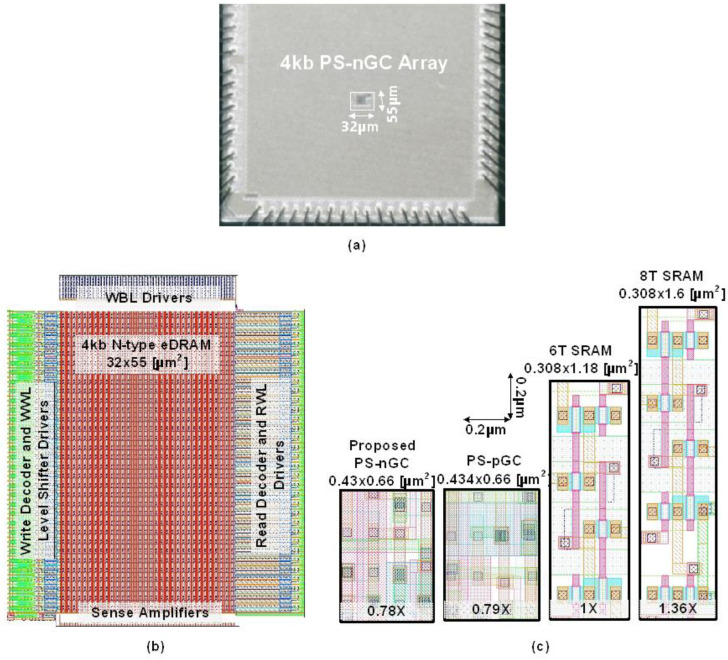
(**a**) Die microphotograph, (**b**) layout of proposed 4 kb eDRAM macro, and (**c**) comparison of layouts of proposed PS-nGC, PS-pGC, 6T SRAM bitcell, and 8T SRAM bitcell.

Figure 15a,b show the die micrograph of the fabricated eDRAM macro in 28 nm CMOS process and the detailed layout design of the 4 kb n-type eDRAM macro, respectively. The implemented eDRAM core occupies an active area of 32 µm × 55 µm (1760 µm^2^). Each cell area of the PS-nGC is 0.43 µm × 0.66 µm (0.284 µm^2^). Compared with 6T SRAM [34] and 8T SRAM [35] implemented in the same 28 nm CMOS process, the area is reduced by 0.78 times and 0.58 times, respectively.

**Figure 16:**
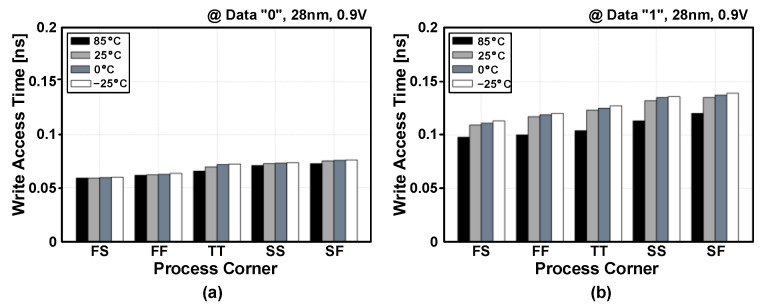
Post-layout simulated write access times of PS-nGC storing data (**a**) “0” and (**b**) “1” across five process corners and four temperature cases.

**Figure 17:**
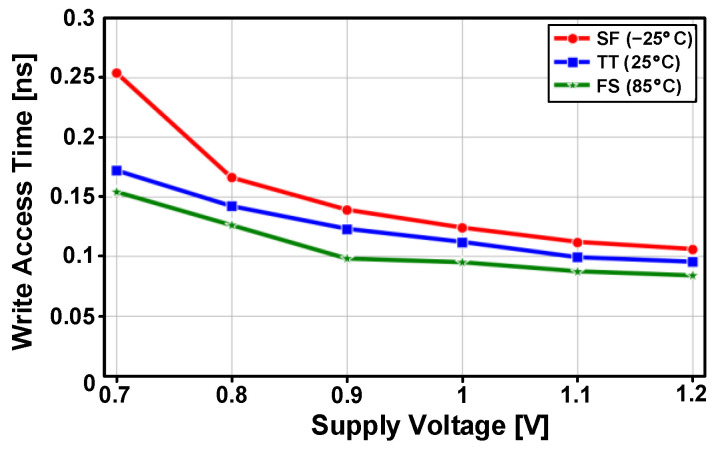
Post-layout simulated write access time versus supply voltage (0.7–1.2 V) with typical (TT, 25 °C), best (FS, 85 °C), and worst (SF, −25 °C) process and temperature corners.

**Figure 18:**
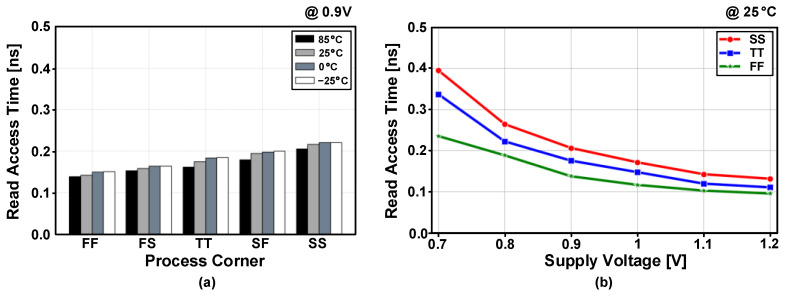
Post-layout simulated read access times (**a**) depending on process corners and temperatures at a supply of 0.9 V and (**b**) across the supply voltage range with typical (TT), best (FF), and worst (SS) process corners and temperature of 25 °C.

The post-layout simulated write access times are shown in Figure 16 for five process corners and four temperature conditions. The PS-nGC can have faster write access times compared to those of the PS-pGC [28]. The eDRAM based on PS-nGC achieved write access times of less than 100 ps for the write operations of data “0” across all process corners and temperature conditions. In case of writing data “1”, the worst access time was 140 ps at the SF process corner and −25 °C. The write access delay times across supply voltage range from 0.7 to 1.2 V and are depicted in Figure 17. The proposed eDRAM achieved write access times shorter than 300 ps across the entire supply voltage range with typical-case (TT, 25 °C), best-case (FS, 85 °C), and worst-case (SF, −25 °C) scenarios. The post-layout simulated read access times of the eDRAM with different process corners and temperature conditions are shown in Figure 18a and include the detection delay by the differential sense amplifier. The eDRAM achieved read access time shorter than 250 ps across all process corners and temperatures at supply voltage of 0.9V. The worst read access times were observed at the SS and SF process corners. Figure 18b shows the simulated read access times within a supply voltage ranging from 0.7 to 1.2 V. Due to the adoption of PS-nGC, the proposed eDRAM can provide a wider operating voltage range than that of the eDRAM with PS-pGC [28]. The low-voltage operation at 0.7 V can further improve the energy efficiency of the eDRAM-based PIM structure.

**Figure 19:**
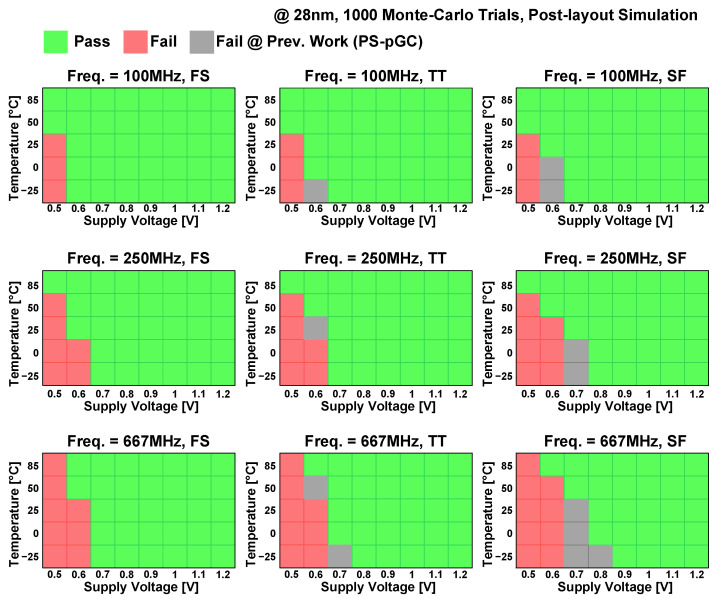
Shmoo plots of proposed PS-nGC: post-layout Monte Carlo simulations (1000 trials) of PS-nGC eDRAM with various operating frequencies (100–667 MHz), process corners (SF, TT, and FS), temperatures (−25 °C to 85 °C), and supply voltages (0.5–1.2 V). Comparison with previous PS-pGC [28] Shmoo plots marked as gray color.

The post-layout Monte Carlo mismatch simulations with 1000 trials were conducted to demonstrate the operation of eDRAM under various operating conditions, as shown in Figure 19. The simulations were conducted under operating frequencies ranging from 100 to 667 MHz; process corners of TT, SF, and FS; supply voltages ranging from 0.5 to 1.2 V; and temperature ranging from −25 to 85 °C. The SF and FS process corners were chosen to evaluate the worst- and best-case scenarios of the write access operations. The results indicated that the eDRAM is capable of operating with supply voltages higher than 0.6 V at an operating frequency of 100 MHz, regardless of process corners and temperatures. At an operating frequency of 250 MHz, the eDRAM can provide normal operation down to a supply voltage of 0.7 V. At the operating frequency of 667 MHz, the eDRAM can operate with an operating voltage ranging from 0.7 to 1.2 V across the entire temperature range and the three process corners. The Shmoo plots in Figure 19 illustrate that the proposed PS-nGC and its 4 kb macro exhibit a wide operating range and high reliability, successfully mitigating the retention time challenges commonly faced in conventional eDRAM macros.

## 5. Comparison between PS-nGC and PS-pGC

In [28], a PS-pGC composed of a 2TAsy gain cell and p-type PSLC was proposed to extend the retention time of eDRAM. It can be implemented with a smaller area compared to 6T and 8T SRAM, while still maintaining data stability in a static manner. However, there were several issues that needed to be addressed in order for it to be adopted in high-performance PIM applications. In this work, to address the issues, the PS-nGC composed of a 2T gain cell and n-type PSLC was presented.

**Figure 20:**
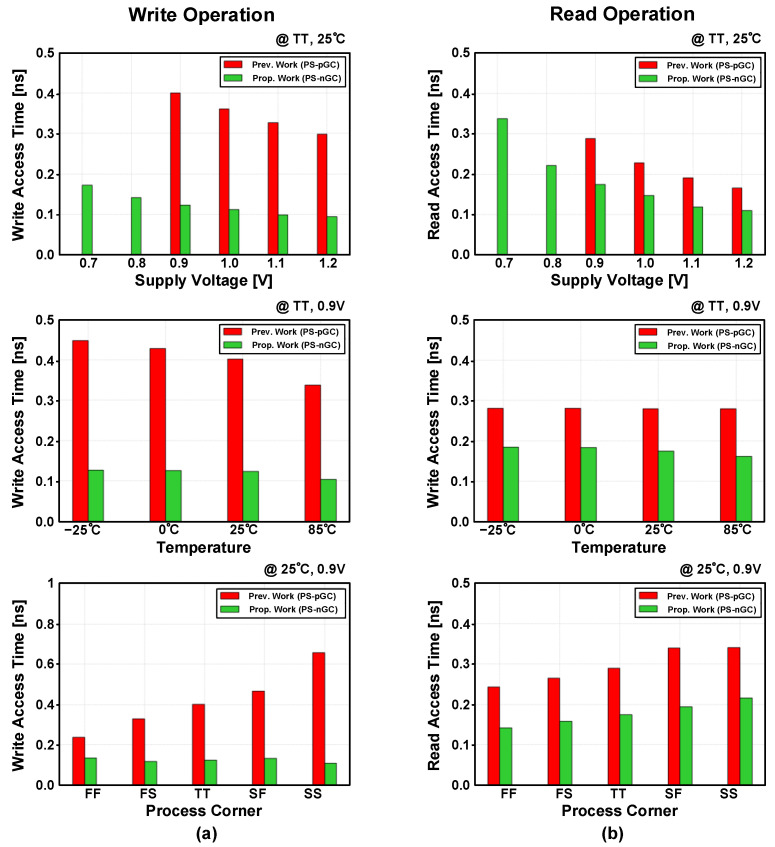
Comparison of (**a**) write access time and (**b**) read access time between previous PS-pGC [28] and proposed PS-nGC under varying temperatures, corners, and voltage simulation conditions.

Figure 20 illustrates a comparison between the previous PS-pGC and the proposed PS-nGC. The PS-nGC achieves a write access time that is 3.27 times faster than that of the PS-pGC. Furthermore, under the same operating conditions, the read access time is about 1.81 times faster than that of the PS-nGC.

Table 1 shows a summarized comparison of the performance between the prior gain cells [24,28,29,30,34,36] and the proposed PS-nGC. The proposed PS-nGC offers not only a compact area enabling higher memory density but also an unlimited retention time eliminating the need for dedicated blocks or power consumption for refresh. Compared with the previous PS-pGC [28], the PS-nGC achieved improved write access time and read access time.

The proposed PS-nGC-based eDRAM macro can be adopted not only for PIMs based on the conventional CMOS process but also for future high-speed switches and accelerators based on plasma or optoelectronic devices. To address the bandwidth limitation of the CMOS process, an all-electronic device based on nanoscale plasma [40] achieved ultrafast switching rates exceeding 10 V per picosecond with a broad power range, surpassing the switching speed of CMOS transistors. Furthermore, extensive exploration of light-based optoelectronic control for high-speed signal processing has been presented [41]. For the next-generation PIMs that may be implemented in the plasma or optoelectronic devices, the PS-nGC can provide not only unlimited retention time but also fast read/write access times, making it suitable for high-speed processing applications.

## 6. Conclusions

This paper presented a PS-nGC of eDRAM architecture for high-speed PIM applications, particularly targeting intelligent sensor hub systems. The PS-nGC leverages 2T gain cell combined with an n-type PSLC circuit. This approach not only ensures unlimited data retention but also significantly enhances both read and write access times. The incorporation of a boosted WWL driving technique has enabled the PS-nGC to operate effectively within a widened supply voltage range, spanning from 0.7 to 1.2 V. Moreover, the active leakage compensation mechanisms integrated into the gain cell, coupled with the improved read and write circuitry, allows the eDRAM macro to achieve resilience to the adverse effects of process, voltage, and temperature variations. The PS-nGC offers a highly compact implementation with a minimal bit cell area of just 0.284 μm^2^. Furthermore, it boasts rapid read and write access times, with the write time being 3.27 times faster and the read time being 1.81 times faster, thereby significantly enhancing high-speed PIM operations. Additionally, as capacitors are not needed for data retention, MOM capacitors can be employed for MAC operations in PIM. These attributes collectively position the PS-nGC and its associated eDRAM macro as a strong foundation for intelligent sensor hub systems.

## Figures and Tables

**Table 1:** Performance summary and comparison with previous works.

	2T [29]	2T [30]	3T [36]	3T [24]	4T [32]	PS-pGC [28]		This Work	
Bitcell Schematic	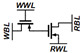	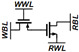	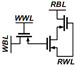	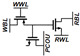	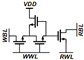	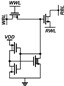		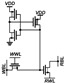	
Process	65 nm	65 nm LP	65 nm LP	65 nm LP	28 nm FD-SOI	28 nm		28 nm	
Bitcell Area	0.275 μm^2^	0.478 μm^2^	0.627 μm^2^	0.674 μm^2^	0.23 μm^2^	0.286 μm^2^		0.284 μm^2^	
Bitcell Area Normalized to 28 nm Process	0.075 μm^2^	0.13 μm^2^	0.21 μm^2^	0.26 μm^2^	0.23 μm^2^	0.286 μm^2^		0.284 μm^2^	
Retention Time	10 μs @ 85 °C	276.5 μs @ 85 °C	1.25 ms @ 85 °C	325 μs @ 85 °C	154 μs @ 85 °C	Static		Static	
Maximum Freq.	2 GHz	667 MHz	NA	1 GHz	66 MHz	100 MHz	667 MHz	100 MHz	667 MHz
*V_DD_* Range	0.7–1.1 V	0.8–1.4 V	0.8–1.3 V	0.8–1.2 V	0.6–0.9 V	0.7–1.2 V	0.9–1.2 V	0.6–1.2 V	0.7–1.2 V
Temp. Range	25–85 °C	25–85 °C	25–85 °C	25–85 °C	0–85 °C	−25–85 °C		−25–85 °C	
Write Access Time	NA	0.21 ns @ 85 °C	0.27 ns @ 85 °C	1.5 ns @ 85 °C	0.46–0.67 ns @ 27 °C	0.34 ns @ 85 °C, TT		0.104 ns @ 85 °C, TT	
Read Access Time	NA	0.46 ns @ 85 °C	0.61 ns @ 85 °C	1 ns @ 85 °C	<3 ns @ 27 °C	0.29 ns @ 85 °C, TT		0.16 ns @ 85 °C, TT	
Additional Bit/Wordline?	No	No	No	Yes	No	No		No	
Need Refresh?	Yes	Yes	Yes	Yes	Yes	No		No	
Retention Power	508 mW/2 Mb @ 85 °C	1.16 mW/Mb @ 85 °C	1.25 mW/Mb @ 85 °C	NA	909 nW/8 kb @ 85 °C	22.5 μW/4 kb @ 85 °C, TT		25.4 μW/4 kb @ 85 °C, TT	

## Data Availability

Data are contained within the article.
